# Lack of association between COVID-19 vaccines and miscarriage onset using a case-crossover design

**DOI:** 10.1038/s41598-024-57880-8

**Published:** 2024-03-27

**Authors:** Irati Gastesi Orbegozo, Lucía Cea-Soriano, Ana Llorente, Consuelo Huerta-Álvarez

**Affiliations:** 1 Biomedical Research Foundation Hospital 12 de Octubre, Instituto de Investigación 12 de Octubre (imas12), Madrid, Spain; 2 Department of Public Health and Maternal Child Health, Faculty of Medicine, Complutense University of Madrid, Pza. Ramón y Cajal, s/n. Ciudad Universitaria, 28040 Madrid, Spain; 3 BIFAP, Division of Pharmacoepidemiology and Pharmacovigilance, Spanish Agency for Medicines and Medical Devices (AEMPS), Madrid, Spain

**Keywords:** COVID-19 vaccine, Miscarriage, Case-crossover study, Pharmacoepidemiology, Epidemiology, Obstetrics, Risk factors, Epidemiology, Outcomes research, Drug development

## Abstract

Pregnant women might have an increased risk of SARS-COV-2 infection. Although evidence towards the efficacy and safety of COVID-19 is growing still there is room for improvement on the knowledge towards pregnancy adverse events, such as miscarriage. We explored the association of COVID-19 vaccine with the risk of miscarriages using the Real-World. We identified a cohort of vaccinated pregnancies using the BIFAP database which contains systematically recorded data on care patients in Spain (N = 4054). We then restricted it to those women who had a miscarriage using a validated algorithm (N = 607). Among them, we performed a case-crossover design to evaluate the effect of intermittent exposures on the risk of miscarriage. Adjusted Odds Ratio with their confidence intervals were calculated using two analytical approaches: conditional logistic regression and Generalized Linear Mixed-Effects Models. A total of 225 (37.1%) were aged 35–39 years. The most common comorbidities were asthma, migraine, gastritis, and hypothyroidism. A total of 14.7% received only one dose of COVID-19 and 85.3% two doses, respectively. A total of 36.8% of women with one dose and 27.6% with two doses received the vaccine 7 days prior to the miscarriage. Corresponding adjusted estimates for the risk of miscarriage using the conditional logistic regression where as follows: 1.65 (95% CI 0.85–3.23) when using as the sum of 3 control moments among women with one dose, 1.02 (95% CI 0.72–1.46) among women with two doses and 1.03 (95% CI 0.72, 1.46) using the whole study population. Very similar results were obtained when conducting the Generalized Linear Mixed-Effects Models. There was no overall increased risk of miscarriage onset associated with COVID-19 vaccine although contradictory results were found according to the number of doses. Further studies are required with larger sample sizes to assess this association.

## Introduction

Miscarriage is the spontaneous loss of a pregnancy before the 20th week. Thus, the death of an embryo or fetus before it can survive independently. There are a lot of causes of miscarriage such as problems that are related to mother’s or father’s genes, bad habits of the parents (drug and alcohol abuse and smoking), exposure to environmental toxins, infection, and hormone problems. Women who know they are pregnant, about 10–25% will have a miscarriage^1^.

Several studies have concluded how pregnant women might have an increased risk of SARS-COV2 infection^2–4^. Current knowledge towards COVID-19 in pregnancy includes a potential increased risk of neonatal and maternal adverse outcomes such as preterm birth, and cesarean section or multisystem inflammatory syndrome^5,6^, a more frequent admission to the intensive care unit (ICU) and respiratory support by mechanical ventilation^7,8^, and a possible, although controversial, vertical transmission^9,10^.

Vaccination during pregnancy, as a primary prevention strategy, has dual benefits for the mother as well as in the prevention of vertical transfer of infection to the fetus. Inactivated vaccines can be safely administered to pregnant women except for live vaccines that are administered under very specific conditions^11^. Currently there are available two types of COVID-19 vaccines: one adenoviral vector vaccine, like the vaccine against flu, and mRNA vaccine. Current recommendations specify that any of the two kinds of vaccines can be administered to pregnant or lactating persons, with no preference for the vaccine type^12^. Although some countries such as the UK potentially recommended mRNA vaccine due to safety concerns towards the cerebral venous sinus thrombosis and other thrombosis with thrombocytopenia episodes observed with the adenovirus vaccine^13^.

In terms of safety focused on pregnancy adverse events, although data has been and still is limited there are reassurance results. For example, the registry study conducted by the Centers for Disease Control and Prevention (CDC; CDC v-safe Covid-19 pregnancy) had followed women up to 3 months after pregnancy completion found how of adverse pregnancy and neonatal outcomes rates (i.e., pregnancy loss, pre- term birth, small for gestational age, and congenital anomalies) were similar to the published background rates^14,15^. Other studies have found similar adverse pregnancy rates than the one observed with the 2009 H1N1 inactivated influenza vaccine. Focusing on the first trimester, a prior study using a Norwegian registry conducted a case control analysis to evaluate the association between COVID 19-vaccine and risk of miscarriage, which concluded there was not a link^16^. Likewise, preliminary results from the CDC v-safe Covid-19 pregnancy registry were consistent with the expected risk of spontaneous abortion^17^. Keeping in mind the endemic behavior of SARS-COV2 and how women may still be vaccinated in the first trimester not being aware of being pregnant, more complete, and specific information about the birth outcomes, especially within the first trimester, are warranted.

To address this knowledge gap, we conducted a case crossover study that allows us to evaluate the effect of intermittent exposures on the risk of acute events. Thus, we explored the association of COVID-19 vaccine with the risk of miscarriages using the Real-World Data from BIFAP (Base de datos para la Investigacion Farmacoepidemiológica en el Ámbito Público) database which contains systematically recorded data on more than ten million primary care patients in Spain.

## Material and methods

### Data source

BIFAP database was used to perform the current study. Briefly, it is a computerized medical longitudinal population-based database of anonymized electronic medical records of primary care practitioners and pediatricians (PCP) from nine participating Autonomous Regions (out of 17) in Spain. It is representative to the Spanish population in terms of age and sex distribution. Data include demo- graphic factors, consultation visits, referrals, hospital admissions, laboratory test results, diagnostic procedures, diagnoses, and prescriptions. Clinical data are recorded using international Classification of Primary Care—Second Edition (ICPC-2) and International Classification of Diseases (ICD-9)^18,19^. And medications, using the ATC classification, are automatically recorded by the PCP or specialists. The study protocol was approved by the BIFAP Scientific Committee (Ref 15_2020) and Ethical Committee on Clinical Research of the Hospital Clínico San Carlos of Madrid (Ref 20/749-E_COVID). BIFAP has been extensively described elsewhere^20^.

### Source population

The study population consisted of all women of childbearing age (15–49 years) during the study period between January 2021 and October 2021 from five regions with SARS Cov-2 data available (the latest date upon the date of conducting the study). To participate in the study, and as an inclusion criterion, women must have been registered with their primary care physician at least 1 year before entering the study. This criterion and time frame (i.e., 1 year) serves to ensure a minimum of information recorded on the patients, and to be able to collect demographic data (lifestyle, such as BMI) and comorbidities, recorded by the physician beforehand. All women with a diagnosis of SARS-CoV-2 before the study entry were excluded.

### Identification of the cohort of pregnant women, gestation time

Once meeting the inclusion criteria, we used an adaptation from a valid algorithm to identify pregnancies designed by the authors and applied in other databases with similar characteristics which includes the gestational age and a pregnancy outcome algorithm, both have been designed and described in detail previously^18^. Briefly, as a first step, the following indicators of pregnancy were identified: (i) indicators of conception, (ii) indicators of end of pregnancy; and (iii) other codes compatible with a pregnancy, such as pregnancy test, prenatal visits, pregnancy complications, etc. After assignment of the validated gestational age, women identified as pregnant were classified according to pregnancy outcome into (i) term pregnancy, (ii) miscarriage or (iii) stillbirth, (iv) unspecific pregnancy. All those women whose gestational age couldn´t be calculated (pregnancy with non-specific gestational age) were excluded.

### Vaccinated cohort and miscarriage episodes

**Figure 1:**
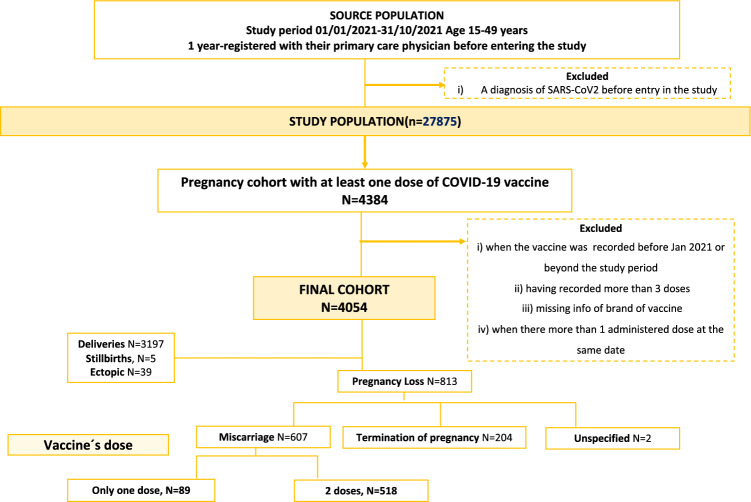
Flow chart of the study design.

Once identified the cohort of pregnancies, we restricted the cohort to those with at least one recorded dose of COVID-19 vaccines within pregnancy, that is from Last menstrual period (LMP) date up to delivery date. Of note, we excluded the following scenarios: (i) when the registry of the vaccine was recorded before Jan 2021, (ii) when the vaccine was recorded beyond the end of pregnancy date, (iii) having recorded more than 3 doses, (iv) when there was missing information on the brand of vaccine and (v) when there were recorded 2 administered doses per vaccine brand at the same date. The total cohort of vaccinated pregnancies encompassed a total of 4054 women. Finally, out of them, we selected all women with a miscarriage episode as the outcome of pregnancy. To do that, first, we subdivided all pregnancy losses into three main categories according to the code and descriptor used to register the episode: miscarriages, terminations of pregnancy (TOP) and unspecified abortions. For each group presented above, in a prior study we randomly selected a sample of medical records of each subcategory and manually reviewed them. All women initially classified as miscarriages were confirmed (positive predictive value > 90%)^19^. Therefore, our final sample of women suffering from miscarriages was 607 (Fig. 1).

### Study design

We conducted a case crossover (CXO) design. Essentially, the principle of this design is to answer the question: “Was the case-patient doing anything peculiar and unusual just before disease onset?” or “Did the patient do anything unusual compared to his routine?”. The assumption is that if there are triggering events, these events should occur more frequently immediately prior to disease onset than at any similar period distant from disease onset^21–25^. The CXO design resembles a matched case–control study where events are fixed, and exposure is random. Control moments are derived from the follow-up time or person-time of the cases before the event occurs. That is, controls are periods of time when the person who developed the event of interest had not yet developed at that time. This provides a set of matched variables corresponding to the event of interest and to control periods that may be analyzed as a matched case–control study^21,25^.

This design uses the difference of the exposure rates just before the event (case) with those at other times (controls) to estimate an odds ratio of the outcome associated with the exposure. Previous studies on the effects on drugs during pregnancy showed the validity of this design^26^. When statistically analyzing this design, normally, a conditional logistic regression model is fitted^21^.

### Exposure definition

When defining the duration of the case–control windows, it was considered that they could not be too long as it was the first months of pregnancy. Therefore, a duration of 1 week was set. COVID-19 vaccination was collected through the active surveillance system implemented during the SARS-CoV-2 pandemic and automatically recorded BIFAP. The surveillance system included systematic recording of COVID-19 cases as well as details in vaccination.

For this study, we performed a case-crossover design with one case moment and three control moments. The case window is defined, within the previous seven-day period, as the risk period for the event (miscarriage). In addition, the control windows of the design are defined within the previous 7–14 days, within the previous 15–21 days and 22–28 days according to miscarriage date and the sum of control moments. Of note, as published elsewhere, using more than one control period per case window or, even using the entire case history would increase the precision of the estimates^26^ (See Supplemental Fig. 1).

### Other variables and potential confounders

We obtained information on patient demographics and on comorbidities any time before the LMP date using the most recent recorded value/status as appropriate. Specifically, for comorbidities we collected: obesity, anemia, hypertension, multiple sclerosis, deep venous thrombosis/pulmonary embolism (DVT/PE), ischemic heart disease (IHD), arrhythmias, depression, asthma, chronic obstructive pulmonary disease (COPD), epilepsy, psoriasis, migraine, diabetes, gastritis, hypothyroidism, and HIV. Medication exposure was defined as a prescription in the 90 days before the LMP date, the most frequently prescribed drugs were collected.

Additionally, specific medications used during pregnancy and potentially associated with increased risk of miscarriage were considered as potential confounders of the case-crossover model and collected at the dates of the event and at each control moment (7, 14, 21 and 28 days before the case). Thus, the following were considered: paracetamol, opioids, anxiolytics, antiplatelets, antimigraine, non-steroidal anti-inflammatory drugs (NSAIDs), Selective serotonin reuptake inhibitors (SSRIs), diuretics, proton pump inhibitors (PPIs), antibiotics, respiratory drugs, thyroid hormone, statins.

### Statistical analyses

The case-crossover (CXO) design’s analysis of these data compared the case moment to the control moments I, and to the sum of the 3 control moments. Additionally, comparison of case moments with control moments 2 and 3 separately were also done. Odds Ratio (OR) were estimated by the ratio of the number of cases exposed only during the case window to the number of cases exposed only during the control window/s. Only discordant pairs contribute to the estimation of the odds ratio in matched analyses; therefore, we would obtain the same estimates by including only the cases exposed during the study. For this design we first used a conditional logistic regression model to estimate OR and 95 percent confidence intervals. Crude model has only the exposure variable (Vaccine) and the adjusted one included potential confounders that change over gestational time of the study, such as potential teratogenic medications (*i.e. paracetamol, opioids, anxiolytics, antiplatelets, antimigraine, Non-Steroidal Anti-inflammatory Drugs (NSAIDs), Selective serotonin reuptake inhibitors (SSRIs), diuretics, Proton Pump Inhibitors (PPIs), antibiotics, respiratory drugs, thyroid hormone, statins)*. Those were added to the model denoting the absence or presence of prescriptions of each separate type of medication listed, within the 7 days prior to the case and each control moments, respectively.

To test the accuracy of our results, we also performed a secondary statistical analysis, including crude and adjusted models by potential confounders using the Generalized Linear Mixed-Effects model (GLMMs). There are several reasons why a researcher might choose to use a GLMM as well for a case-crossover study. First, GLMMs can handle correlated data more efficiently than traditional regression models, such as logistic regression. Second, GLMMs can handle missing data and unbalanced data, which are common issues in case-crossover studies. Finally, GLMMs can provide more accurate estimates of the exposure-outcome association when there is within-individual variation in exposure over time, which is a key characteristic of case-crossover studies. All statistical analyses were conducted using statistical software R version 4.1.1

### Institutional review board statement

The study protocol was approved by the BIFAP Scientific Committee (reference 15_2020_MOD) and the Ethical Committee on Clinical Research of the Hospital Clínico San Carlos of Madrid (reference 20/749-E_COVID). Authors confirm that all methods were performed in accordance with the relevant guidelines and regulations.

### Informed consent statement

BIFAP meet with the Spanish Data Protection Law (https://www.boe.es/eli/es/lo/2018/12/05/3/con and BIFAP`s Data Governace http://bifap.aemps.es/docs/Gobernanza_acceso_datos_BIFAP_v2_2021.pdf ), under this condition, secondary use of anonymized data, informed consent was not required. Under Spanish regulation ethics committee was mandatory.

## Results

### Characteristics of the cohort study population

We identified a total of 607 women with a miscarriage and vaccinated and at least one dose of the COVID-19 vaccine. Table 1 shows the main baseline characteristics of the study population. A total of 225 (37.1%) and 148 (24.4%) were aged 35–39 and 30–34 years respectively. The most common comorbidities are asthma, migraine, gastritis, and hypothyroidism. There were 65 (10.7%) women with asthma, 78 (12.9%) with migraine, 45 (7.4%) with gastritis and 48 (7.9%) with hypothyroidism. A total of 41 (6.8%) were obese. Less common comorbidities were IHD (0.2%), COPD (0.2%), VIH (0.2%), multiple sclerosis (0.3%) and DVT/PE (0.3%). In terms of drug utilization, the most used drugs during pre-pregnancy were NSAIDs, antibiotics and anxiolytics. Specifically, the prevalence of use was as follows: 75 (12.4%) for NSAIDs, 68 (11.2%) for antibiotics and 39 (6.4%) for anxiolytics, respectively (Table 1).

**Table 1:** Baseline characteristics of study population.

	Cases (n = 607)
Age, n (%)	
< 25 years	32 (5.3%)
25–29 years	62 (10.2%)
30–34 years	148 (24.4%)
35–39 years	225 (37.1%)
40 and more	140 (23.1%)
Obesity, mean (SD)	41 (6.8)
Comorbidities, n (%)	
Anemia	11 (1.8%)
Hypertension	18 (3.0%)
Multiple sclerosis	2 (0.3%)
DVT/PE	2 (0.3%)
IHD	1 (0.2%)
Arrhythmia	22 (3.6%)
Depression	38 (6.3%)
Asthma	65 (10.7%)
COPD	1 (0.2%)
Epilepsy	2 (0.3%)
Psoriasis	4 (0.7%)
Migraine	78 (12.9%)
Diabetes	11 (1.8%)
Gastritis	45 (7.4%)
Hypothyroidism	48 (7.9%)
VIH	1 (0.2%)
Drug utilization, n (%)	
Paracetamol	32 (5.3%)
Opioids	16 (2.6%)
Anxiolytics	39 (6.4%)
Antiplatelets	7 (1.2%)
Antimigraine	6 (1.0%)
NSAIDs	75 (12.4%)
SSRIs	14 (2.3%)
Diuretics	3 (0.5%)
PPIs	28 (4.6%)
Antibiotics	68 (11.2%)
Respiratory drugs	33 (5.4%)
Thyroid hormone	33 (5.4%)
Statins	3 (0.5%)
Antidepressants	26 (4.3%)

*IHD ischemic heart diseases, *COPD* Chronic obstructive pulmonary disease, *DVT/PE* deep vein thrombosis/pulmonary embolism, *HIV* human immunodeficiency virus, *NSAIDs* non-steroidal anti-inflammatory drugs, *SSRIs* selective serotonin reuptake inhibitors, *PPIs* proton pump inhibitors.

### Distribution of the date of vaccination according to gestational age

The distribution of the date of vaccination according to gestational age among women who received only one dose and women who received two doses, separately, is shown in Supplementary Figs. 2–3. There was no trend on the prescription of COVID-19 vaccines according to gestational age, the distribution of vaccines was heterogeneous according with number of doses. While the distribution of vaccines among women receiving only one dose (N = 89, 14.7%) was more concentrated within the first 12th weeks there was a more homogeneous distribution among women receiving two doses (N = 518, 85.4%).

### Distribution of COVID-19 vaccine and risk and control moments

Table 2 shows the distribution of exposure to COVID-19 vaccines (one or two doses) according to brand and according to case and control moments. Among women receiving only one dose (N = 89, 14.7%), the vast majority of women received an RNA vaccine encompassing 98% of women, corresponding distribution were as follows: 59.6% for Pfizer, 21.4% for Janssen and 18% for Moderna. Among women receiving two doses (N = 518) only 5 women received a combination between vaccine´s brand, focusing on remaining women (N = 513) we found similar distributions than the observed in women receiving only one dose, mRNA vaccine encompassed a total of 90% of all vaccinations, a total of 73% corresponded to Pfizer and 16.6% to Moderna (Table 2).

**Table 2:** Distribution of exposure to COVID-19 vaccines according to brand.

According to Brand	Only one dose N = 89		Two doses, N = 518	
	N	%	N	%
AstraZeneca	1	1.12	50	9.65
Janssen	19	21.35	1	0.19
Moderna	16	17.98	86	16.60
Pfizer	53	59.55	381	73.55
Total vaccines	89	100	518	100

In terms of case and control moments, the population was distributed in 4-time windows, as described in the study design section. Among women receiving only one dose (N = 89), 15.7% fell in the case window (that is exposed within the 7 days prior to miscarriage onset) and 26.9% within any control moment (from I to III). The proportion of women exposed during the case moment among women receiving two doses (N = 518) was 12.7% while 33.4% fell within any control moment (from I to III) (Table 3).

**Table 3:** Distribution of exposure to COVID-19 vaccines according to case and control moments.

Number of doses falling in each case and control moment window	Only one dose N = 89		Two doses, N = 518	
	N	%	N	%
Case moment	14	15.73	66	12.74
Control moment I	6	6.74	56	10.81
Control moment II	6	6.74	61	11.77
Control moment III	12	13.48	56	10.81

Numbers do not sum the total denominator as not all doses fell into that specific time windows.

### Association of exposure to COVID-19 vaccine with miscarriage onset

The lines that follow correspond to the association between COVID-19 and miscarriage onset stratifying the study population into two subgroups: women who received only one dose (N = 89) and women who received two doses (N = 518) and the whole study cohort including receiving either one or two doses (N = 607).

### Women receiving only one dose

**Figure 2:**
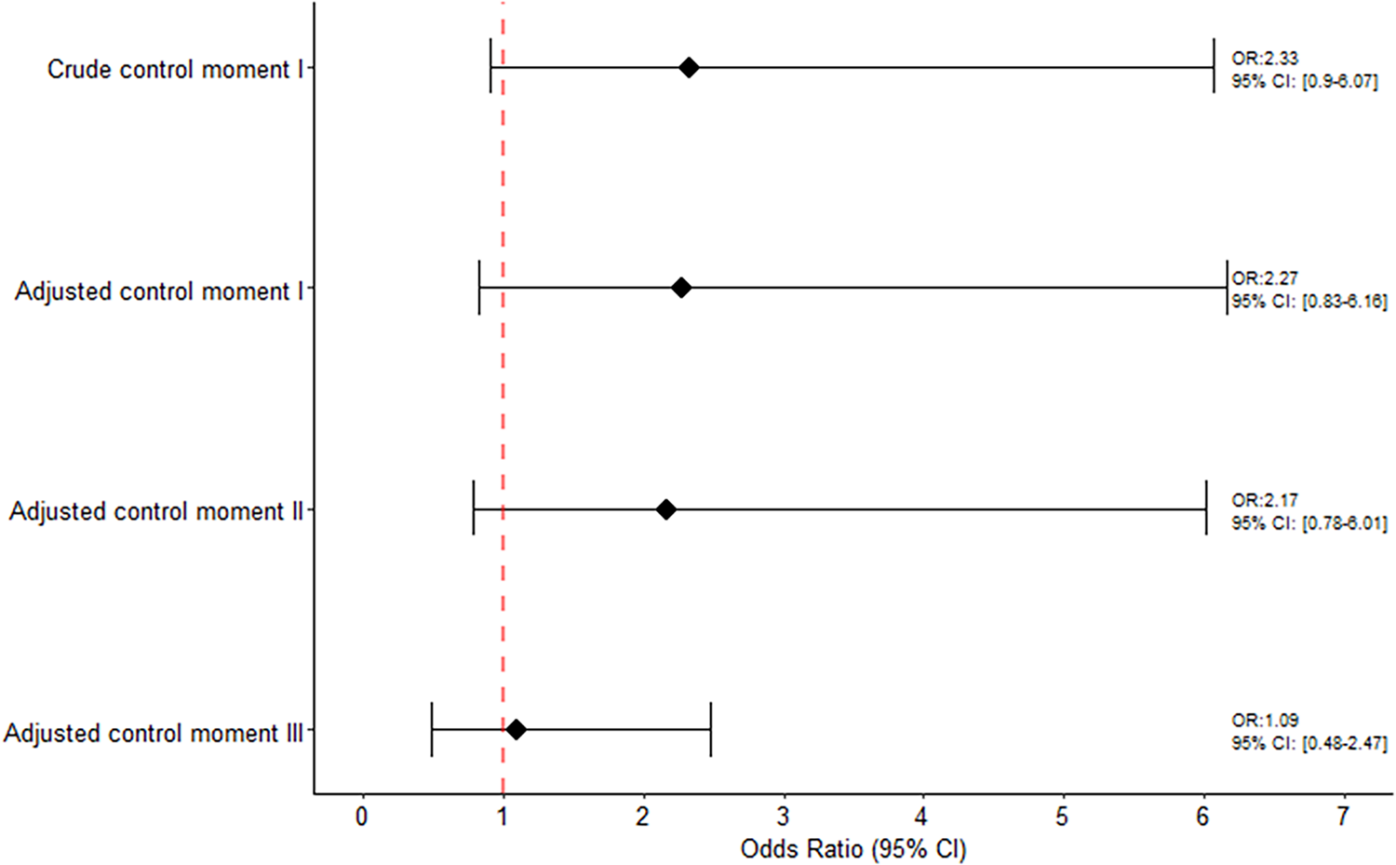
Association between COVID-19 vaccine and risk of miscarriage among women receiving only one dose.

The association between COVID-19 and miscarriage onset is illustrated in Fig. 2. Focusing on the results obtained using the conditional logistic regression we did not observe an increased risk of miscarriage onset associated with COVID-19 vaccine. The crude ORs of miscarriage using the sum of control moments was 1.75 (95% CI 0.91–3.38) and 1.65 (95% CI 0.85–3.23) for the adjusted estimate (data not shown). Specifically, corresponding adjusted estimates when using each control moment window were: 2.27 (95% CI 0.83–6.16) when using control moment I as reference, 2.17 (95% CI 0.78–6.01) when using control moment II as reference, and 1.09 (95% CI 0.48–2.47) using control moment III as reference, respectively.

Similar results were found when conducting the Generalized Linear Mixed-Effects Models. The crude ORs of miscarriage using the sum of control moments was 1.89 (95% CI 0.91–3.80) and 1.88 (95% CI 0.92–3.86) for the adjusted estimate (data not shown). Specifically, corresponding adjusted estimates when using each control moment window were: 2.58 (95% CI 0.91–7.34) when using control moment I as reference, 2.80 (95% CI 1.00–7.89) when using control moment II as reference and 1.07 (95% CI 0.45–2.54) using control moment III as reference (Supplemental Fig. 4).

### Women receiving two doses

**Figure 3:**
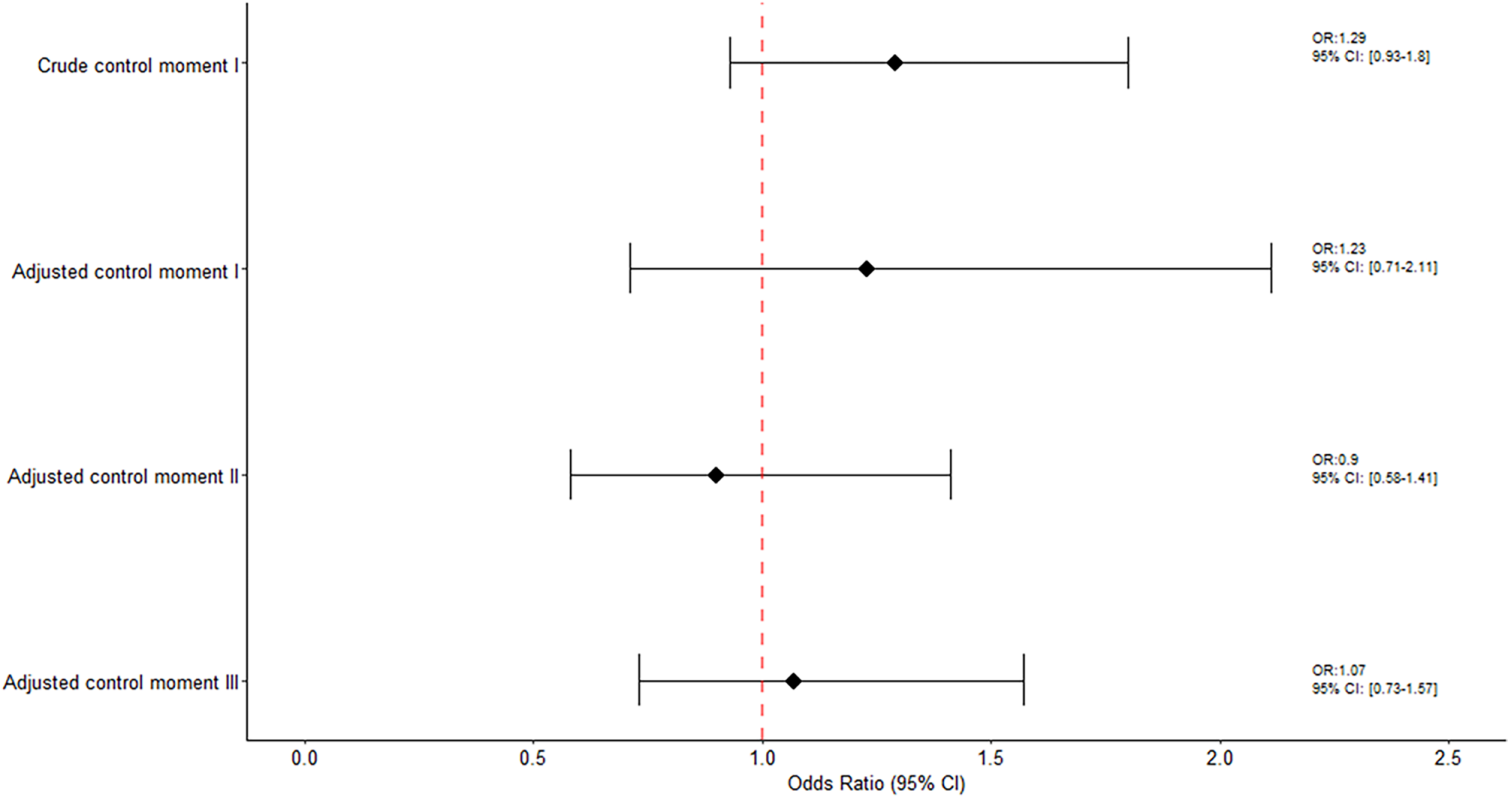
Association between COVID-19 vaccine and risk of miscarriage among women receiving two doses.

Among women receiving two doses and taking the closest administration dose to miscarriage onset, the crude ORs of miscarriage using the sum of control moments was 1.15 (95% CI 0.86–1.52) and 1.02 (95% CI 0.72–1.46) for the adjusted estimate. Specifically, corresponding adjusted estimates when using each control moment window were: 1.23 (95% CI 0.71–2.11) when using control moment I as reference, 0.90 (95% CI 0.58–1.41) when using control moment II as reference and 1.07 (95% CI 0.73–1.57) using control moment III as reference (Fig. 3).

No association was also found when conducting the Generalized Linear Mixed-Effects Models. The crude ORs of miscarriage using the sum of control moments was 1.17 (95% CI 0.86–1.58) and 1.04 (95% CI 0.71–1.52) for the adjusted estimate. Specifically, corresponding adjusted estimates when using each control moment window were: 1.26 (95% CI 0.72–2.20) when using control moment I as reference, 0.87 (95% CI 0.54–1.40) when using control moment II as reference and 1.08 (95% CI 0.69–1.68) using control moment III as reference. (Supplemental Fig. 5).

### All women receiving either one or two doses

**Figure 4:**
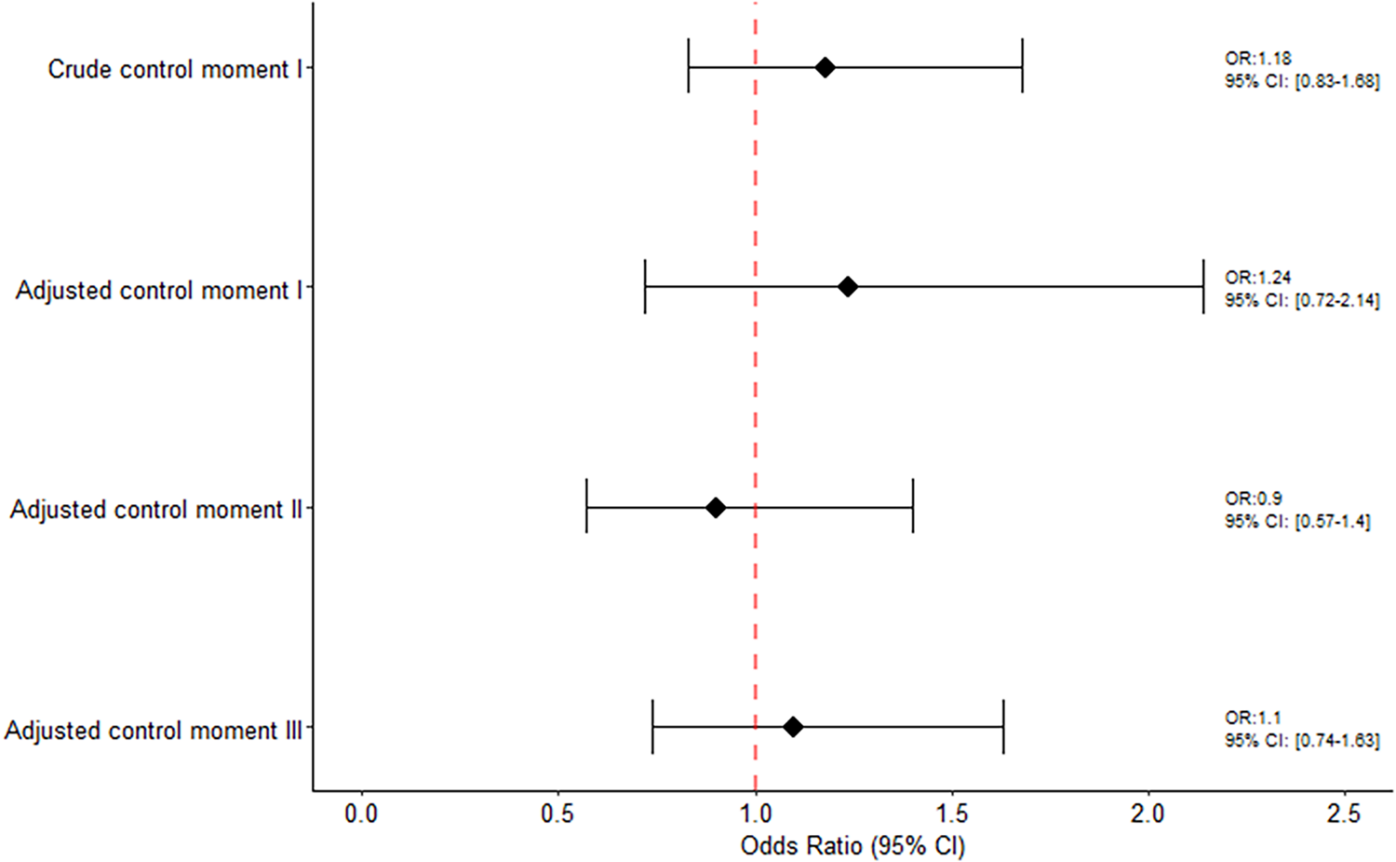
Association between COVID-19 vaccine and risk of miscarriage among women receiving at least one dose.

Among all women encompassing our study regardless of the number of doses received, the crude OR of miscarriage using the sum of control moments was 1.22 (95% CI 0.94–1.58) and 1.03 (95% CI 0.72–1.46) for the adjusted estimate. Specifically, corresponding adjusted estimates when using each control moment window were: 1.24 (95% CI 0.72–2.14) when using control moment I as reference, 0.90 (95% CI 0.57–1.40) when using control moment II as reference and 1.10 (95% CI 0.74–1.63) using control moment III as reference (Fig. 4).

Very similar results were observed when conducting the Generalized Linear Mixed-Effects Models. The crude ORs of miscarriage using the sum of control moments was 1.25 (95% CI 0.95–1.65) and 1.05 (95% CI 0.72–1.53) for the adjusted estimate. Specifically, corresponding adjusted estimates when using each control moment window were: 1.27 (95% CI 0.73–2.21) when using control moment, I, as reference, 0.88 (95% CI 0.55–1.42) when using control moment II, as reference and 1.06 (95% CI 0.68–1.65) using control moment III, as reference, respectively (Supplemental Fig. 6 presents the Generalized Mixed Models).

## Discussion

Among a cohort of 4054 pregnant women with at least one COVID-19 vaccine we identified all women who suffered a miscarriage. The overall prevalence of receiving the COVID-19 vaccine within the week prior to the miscarriage date ranged from 28 to 37%, respectively. Risk of miscarriages was not observed when case vs control moments were compared neither using the sum of control moments non-evaluating control moments separately. Several stratified analyses were performed in order to evaluate the consistency of our results including stratifying our population according to number of doses received and conducting two different analyses approaches: the first one conducting a conditional logistic regression and the second one Generalized Linear Mixed-Effects Models, both reporting crude and adjusted estimates. The results of the stratified analyses were contradictory, while there was a trend towards an increased risk of miscarriage among women receiving only one dose, no association was found for women receiving two doses. The results associated with receiving two doses could be affected by selection bias “survival bias” and thus diluting the true effect. Although these results should be interpreted with caution due to low numbers, further studies are warranted in order to clarify the true association. Data of safety perinatal endpoints associated with COVID-19 vaccine is still scarce in Spain, especially since pregnant women have been excluded from many COVID-19 trials^27,28^ including not only clinical and COVID-19 management but also related to COVID-19 vaccines, enhancing a gap in knowledge towards safety of COVID-19 treatments, and also immunization campaigns. Currently, there are at least four clinical trials of various SARSCoV-2 vaccines in pregnancy undergoing and main results are expected^29–32^. A large number of studies over decades have investigated the role of safety of vaccines during pregnancy. For example, results from several study designs including meta-analysis have concluded how there is no evidence of an increased risk of adverse pregnancy outcomes following influenza vaccination in pregnancy^33–35^. For other types of vaccines such as quadrivalent human papillomavirus vaccine evidence also is reassuring. The results concluded how there is no apparent association between number of doses and timing of administration exposure and miscarriage onset^36^.

Focusing on COVID-19 vaccines, evidence on the benefit–risk profile is still scarce. In terms of safety events on animals receiving a COVID-19 vaccine before or during pregnancy, results did not show any safety concerns^12^. Observational studies, including retrospective and prospective cohorts, and case–control studies, did not find any evidence towards the increased risk of safety issue although the sample size on this study was small^37–39^. As preliminary results, registry studies on COVID-19 mRNA vaccines have reported pregnancy loss rates similar to those prior to the pandemic, ranging from 9 to 17%^14^. In addition, several studies have found no association between^40–43^ vaccinated women and risk of miscarriage compared with unvaccinated controls (ranging the estimates from 1.05 (95% CI 0.78–1.40) up to 3.34 (95% CI 0.37–30.10)) concluding that neonatal outcomes, specifically miscarriages, are not associated with COVID-19 vaccination. Finally, current results coming from systematic reviews and metaanalysis did not find an increased risk of miscarriage and other adverse events and found a protective association with stillbirth^44^. It should be noted that the no major side-effects associated with COVID-19 vaccine was observed especially during the second and third trimester of pregnancy and during breastfeeding^45,46^. Although no association was found, a study using data from surveillance system found similar rates of adverse outcomes in pregnancy than the expected, although it should be noted that the most observed event was spontaneous abortion^47^ It has been thought that the reactivity of SARS-CoV-2 spike protein antibodies with human syncytin-1 protein in trophoblastic tissue might cause placental damage and early pregnancy loss due to the potential homology although further characterization observed low homology which might play no effect in the placenta leading to controversial hypothesis^48–50^. In our study the vast majority of women (> 90%) received mRNA vaccines compared to Adeno-viral based vaccines, in line with other studies^51^. A prior study reported no adverse signals associated with mRNA vaccine and miscarriage rate^52^ however, safety data on adenovirus vector vaccines were still limited. Although the results of the current study are reassuring, in part, further studies with a large sample size with different methodological approaches are needed to warrant the role of safety of vaccines towards miscarriage. Currently, COVID-19 vaccines are recommended for women who are pregnant, to both prevent maternal morbidity and perinatal outcomes, for women who are trying to become pregnant or who might become pregnant in the future^53^. Of note, in addition to maternal benefits, there are neonatal and also infant benefits due to the transplacental transfer of SARS-CIV-2 antibodies. However, several studies have shown a low acceptance of COVID-19 vaccination, with a proportion of acceptance of COVID-19 vaccination during pregnancy of less than 25%^51,54^. Among risk factors associated with willingness to receive the COVID-19 vaccine are the following ones: education, being employed full time, pre-established diabetes mellitus among others^55^. Further studies on women's behavior and decision towards receiving the COVID-19 vaccine will improve current vaccination and further epidemic problems.

Our current research compared the results of this analysis using two different analytical approaches: The first one, the conditional logistic regression model which is extensively applied in case-crossover, and second one the Generalized Linear Mixed-Effects Model. As shown in the results, it has been proven that the results of both analyses did not differ. There was still a slight difference in the results across the analytical approaches. When evaluating all the models with Akaike information criterion (AIC) and Bayesian information criterion (BIC) we observed how conditional logistic regression model offers the best fit, as expected^56–59^.

## Strengths and limitations

In general, the greatest advantage of case-crossover designs is the control for the effect of unmeasured confounding variables, including those with special attention to miscarriage onset such as chromosomal abnormalities accounting for approximately 80% of all pregnancy losses, leading to an automatic control, by design, of any possible confounders. Additionally, the case-crossover design is carried out only with cases reducing time more than other types of designs. With regards to the requirements of the design, the onset of the event, in this case miscarriage, is acute, and exposure to COVID-19 vaccine could be considered as intermittent. The effect of the COVID-19 vaccine is transient and not carry over effect is expected. With regards to control periods, the distribution of the COVID-19 vaccine was almost the same for each period. Potential time-varying confounders considered in the adjusted analysis, such as medication prescribed, did not change results, additional analytical approach either.

Limitations of the design deserve some discussion. By design, we only could focus on short term effects. Data on long term effects on maternal and neonatal outcomes such outcomes after birth is lacking in the current study in part as currently is not possible to link mother to infant records in BIFAP. The duration and timing of control windows is a key design decision, and it depends on the definition of at-risk time^27^. This could be also crucial for this study in which a short period for an at-risk window was assumed as the plausible duration of induction times between the COVID-19 vaccine and the miscarriage.

Although there is still lack of knowledge towards the COVID-19 vaccine on perinatal effects, the results of the current study did not show an increased risk of miscarriages associated with COVID-19 vaccine although stratified analysis by number of doses lead to contradictory results.Future studies are required with larger sample size to assess the association of the exposure COVID-19 vaccine with miscarriage.

### Supplementary Information

Supplementary Figure 1.

Supplementary Figure 2.

Supplementary Figure 3.

Supplementary Figure 4.

Supplementary Figure 5.

Supplementary Figure 6.

## Data Availability

Cea-Soriano remains custodian of the individual patient-level health information. Due to data protection regulations and BIFAP´s data governance, individual level data from this study and administrative registries cannot be shared by the authors, therefore, requests to access the Spanish datasets should be directed to http://www.bifap.org/. The data that support the findings of this study are available from the corresponding author upon reasonable request. Some data may not be made available because of privacy or ethical restrictions.
