# Supplementary figures and images for: Lack of association between COVID-19 vaccines and miscarriage onset using a case-crossover design
Source: Sci Rep. 2024 Mar 27;14:7275. doi: 10.1038/s41598-024-57880-8 (PMC10973422; doi:10.1038/s41598-024-57880-8)

**Supplemental Figure 1.** Ascertainment of exposure according to case and control moments

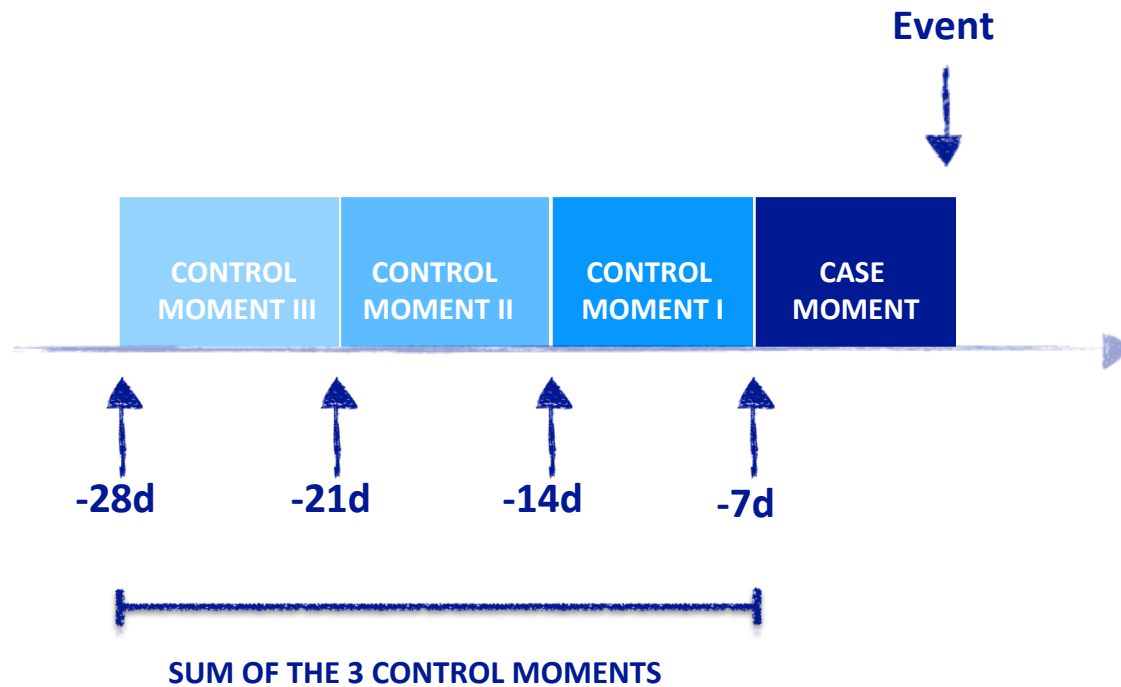

Supplement: Supplementary file 1 — Supplementary Figure 1. [file 41598_2024_57880_MOESM1_ESM.pdf]

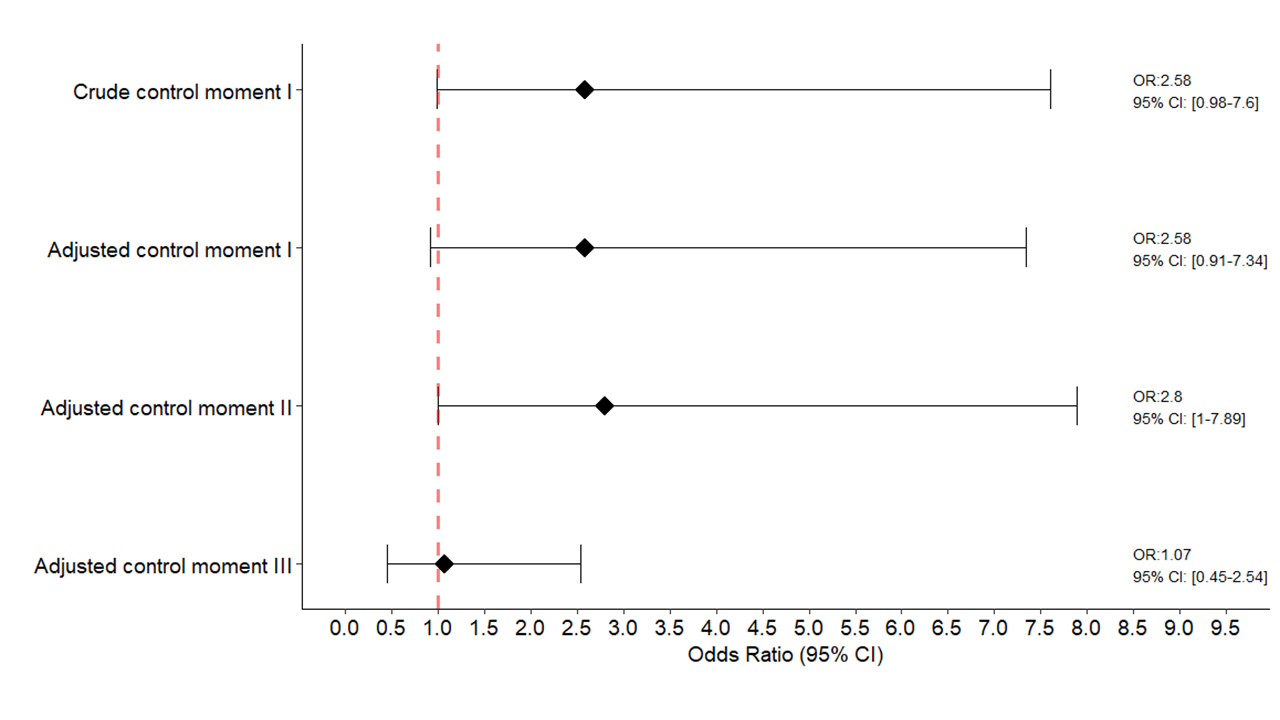

Supplement: Supplementary file 4 — Supplementary Figure 4. [file 41598_2024_57880_MOESM4_ESM.png]

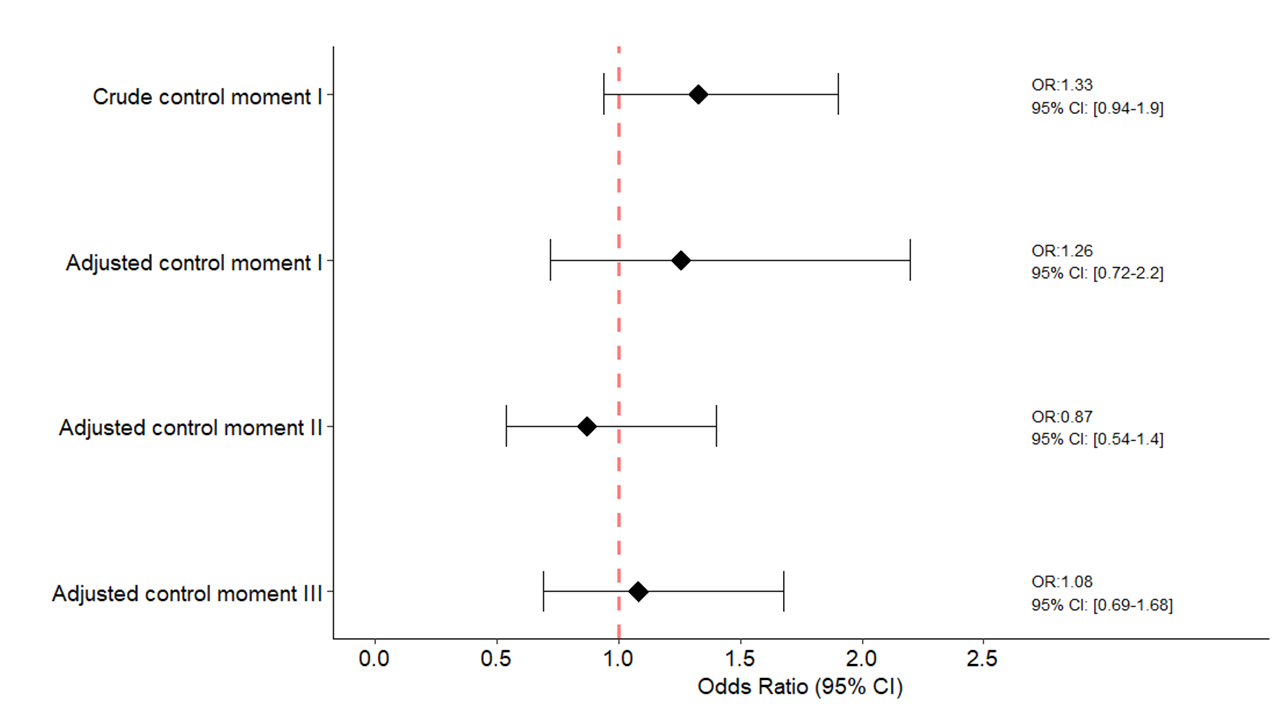

Supplement: Supplementary file 5 — Supplementary Figure 5. [file 41598_2024_57880_MOESM5_ESM.png]

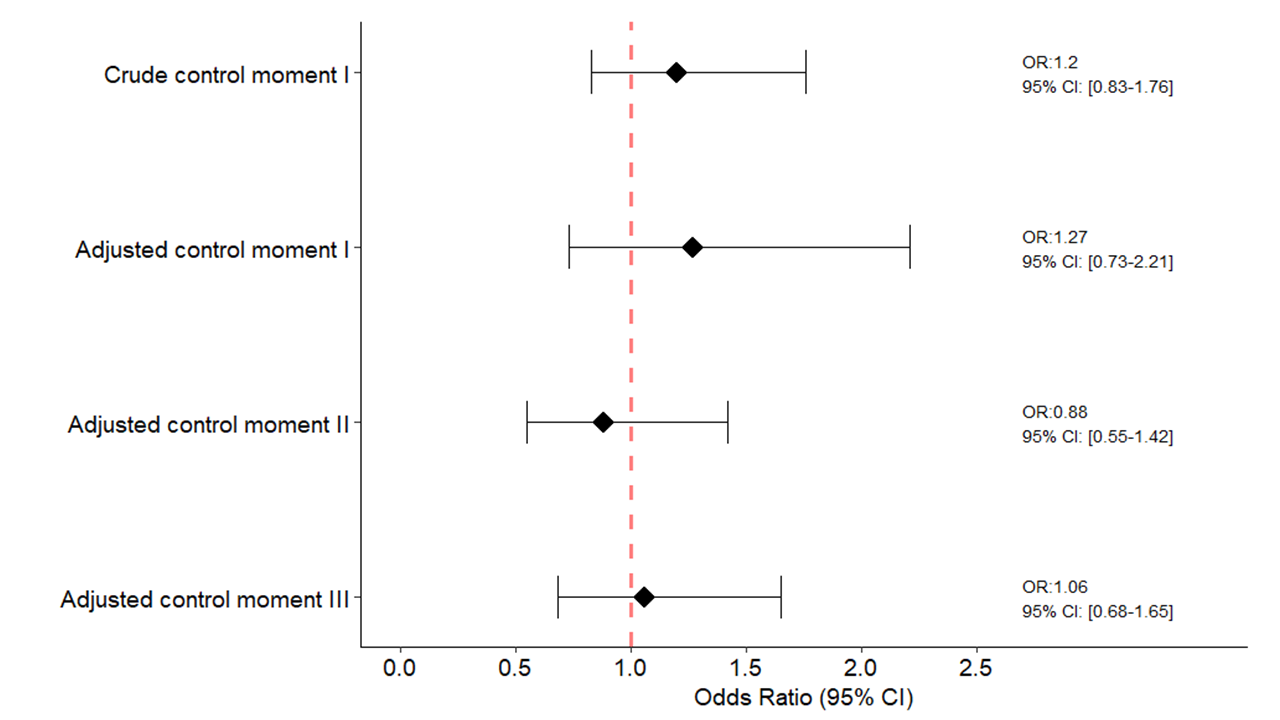

Supplement: Supplementary file 6 — Supplementary Figure 6. [file 41598_2024_57880_MOESM6_ESM.png]
