# Supplementary material for: Lack of association between COVID-19 vaccines and miscarriage onset using a case-crossover design
Source: Sci Rep. 2024 Mar 27;14:7275. doi: 10.1038/s41598-024-57880-8 (PMC10973422; doi:10.1038/s41598-024-57880-8)

Supplemental Figure 2. Distribution of the date of vaccination according to gestational age among women who received only one dose

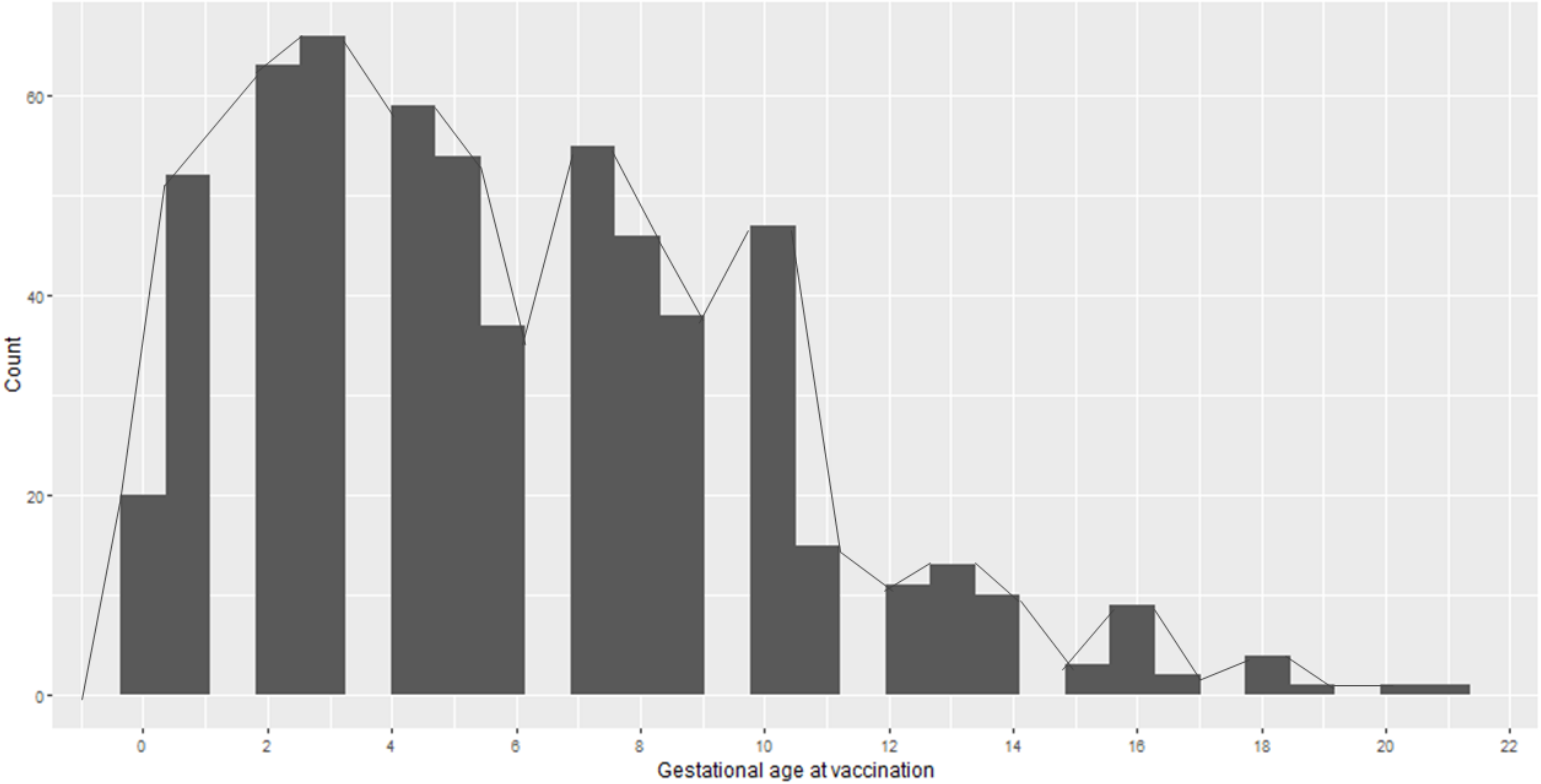

Supplement: Supplementary file 2 — Supplementary Figure 2. [file 41598_2024_57880_MOESM2_ESM.pdf]
