# Supplementary material for: Lack of association between COVID-19 vaccines and miscarriage onset using a case-crossover design
Source: Sci Rep. 2024 Mar 27;14:7275. doi: 10.1038/s41598-024-57880-8 (PMC10973422; doi:10.1038/s41598-024-57880-8)

Supplemental Figure 3. Distribution of the date of vaccination according to gestational age among women who received two doses

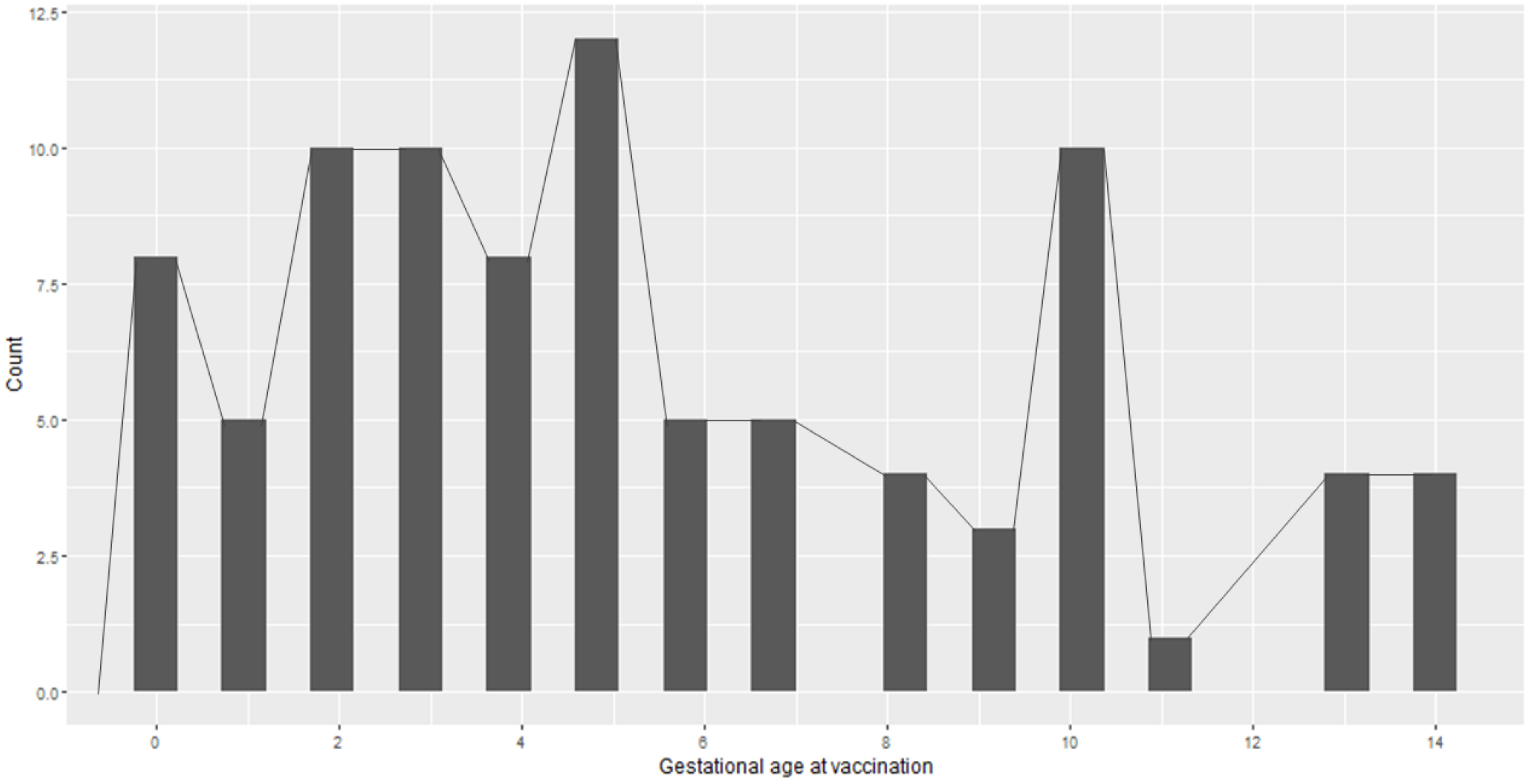

Supplement: Supplementary file 3 — Supplementary Figure 3. [file 41598_2024_57880_MOESM3_ESM.pdf]
